# Comparison of two dengue NS1 rapid tests for sensitivity, specificity and relationship to viraemia and antibody responses

**DOI:** 10.1186/1471-2334-10-142

**Published:** 2010-05-28

**Authors:** Vianney Tricou, Hang TT Vu, Nhu VN Quynh, Chau VV Nguyen, Hien T Tran, Jeremy Farrar, Bridget Wills, Cameron P Simmons

**Affiliations:** 1 Oxford University Clinical Research Unit, Hospital for Tropical Diseases, 190 Ben Ham Tu, District 5, Ho Chi Minh City, Viet Nam; 2 Hospital for Tropical Diseases, 190 Ben Ham Tu, District 5, Ho Chi Minh City, Viet Nam; 3 Centre for Tropical Medicine, University of Oxford, Oxford, United Kingdom. OX3 7LJ, UK

## Abstract

**Background:**

Dengue is a major public health problem in tropical and subtropical countries. Rapid and easy diagnosis of dengue can assist patient triage and care-management. The detection of DENV NS1 on rapid lateral flow tests offers a fast route to a presumptive dengue diagnosis but careful evaluations are urgently needed as more and more people use them.

**Methods:**

The sensitivity and specificity of the Bio-Rad NS1 Ag Strip and SD Dengue Duo (NS1/IgM/IgG) lateral flow rapid tests were evaluated in a panel of plasma samples from 245 Vietnamese patients with RT-PCR confirmed dengue and 47 with other febrile illnesses.

**Results:**

The NS1 rapid tests had similar diagnostic sensitivities (respectively 61.6% and 62.4%) in confirmed dengue cases but were 100% specific. When IgM/IgG results from the SD Dengue Duo were included in the test interpretation, the sensitivity improved significantly from 62.4% with NS1 alone to 75.5% when NS1 and/or IgM was positive and 83.7% when NS1 and/or IgM and/or IgG was positive. Both NS1 assays were significantly more sensitive for primary than secondary dengue. NS1 positivity was associated with the underlying viraemia as NS1-positive samples had a significantly higher viraemia than NS1-negative samples.

**Conclusions:**

These data suggest that the NS1 test component of these assays are highly specific and have similar levels of sensitivity. The IgM parameter in the SD Duo test improved overall test sensitivity without compromising specificity. The SD Dengue Duo lateral flow rapid test deserves further prospective evaluation in dengue endemic settings.

## Background

Dengue is a major public health problem in tropical and subtropical countries [1,2]. Each of the four serotypes of dengue virus (DENV-1 to DENV-4) is able to cause disease. Clinically apparent disease varies in severity from mild undifferentiated fever through to more severe syndromes, such as dengue hemorrhagic fever (DHF) and dengue shock syndrome (DSS). DHF is a vasculopathy characterized by capillary leakage and haematological deregulation, which in severe cases can result in life-threatening hypovolemic shock (DSS). There are no licensed vaccines or specific therapies for dengue, and patient management relies on good supportive care.

There are several reasons why early and accurate diagnosis of dengue is important. First, an early and accurate diagnosis can assist in patient management by directing clinical attention to the appearance of major warning signs of severe or even life threatening complications, e.g. rapidly rising hematocrit, poor peripheral perfusion. Second, an accurate dengue diagnosis prevents unnecessary and possibly expensive antibiotic usage. Third, prompt diagnosis of index cases can facilitate vector control activities in the community so as to mitigate further transmission. Lastly, the expanded use of accurate dengue diagnostics provides important data on the epidemiology and health burden of dengue and in doing so can inform and guide public health policy, particularly as dengue vaccines and anti-virals make their way through development pipelines.

Commercial ELISA tests that detect the DENV NS1 protein in plasma/sera have provided a new avenue for diagnosing dengue [3-10]. The detection of NS1 on rapid lateral flow point-of-care tests offers an even faster route to a presumptive dengue diagnosis [11]. As more point-of-care rapid diagnostic tests (RDT) for dengue, particularly those targeting NS1, reach the marketplace their prices will likely drop to the point they are affordable for use in even resource limited health-care settings. The example of RDTs for malaria that target HRP2 provides a useful example of a point-of-care antigen-detection test where a large number of manufacturers in the market have resulted in cheaper tests such that they are being promoted in even the most resource limited settings [12].

The purpose of the current study was to compare the sensitivity and specificity of 2 commercially available, lateral-flow dengue RDTs (Bio-Rad NS1 Ag Strip and SD Dengue Duo) in a panel of plasma samples from dengue patients with different viral serotypes and viraemia levels. The SD Dengue Duo is distinguished from the Bio-Rad NS1 Ag Strip in that it also tests for DENV IgM and IgG.

## Methods

### Patient samples

The panel of plasma samples used in this study was from patients enrolled in the DENCO study, a multi-centre descriptive study of dengue conducted at the Hospital for Tropical Diseases, Paediatric Hospital #1 and Paediatric Hospital #2 in Ho Chi Minh City, Viet Nam from August 2006 to May 2007. Following written informed consent by the study participant, or a parent/guardian in the case of children, patients above 6 months of age with clinically suspected dengue and fever for less than 7 days were enrolled in the study. Ethical approval was obtained from the Ethics Review Committee of the Hospital for Tropical Diseases, Paediatric Hospital #1 and #2. Two plasma or sera samples were collected from each patient, one at day of enrolment and the second 7-14 days after fever onset.

### Dengue diagnostics

All of the plasma samples used in the panel described in this study were RT-PCR positive for dengue virus using an assay described previously  [11] . IgM and IgG antibody capture ELISA (MAC and GAC ELISA), using DENV/JEV antigens and mAb reagents provided by Venture Technologies (Sarawak, Malaysia), were performed as previously described [13]. The interpretation of primary and secondary serological responses was based on the magnitude of IgG ELISA units in early convalescent plasma samples taking into account the day of illness. The cut-off in IgG ELISA units for distinguishing primary from secondary dengue by day of illness was calibrated using a panel of acute and early convalescent sera from Vietnamese dengue patients that were assayed at the Centre for Vaccine Development, Mahidol University, Bangkok, Thailand using a reference IgM and IgG antibody capture ELISA described previously [14]. DENV loads in plasma were measured using an internally-controlled, serotype-specific, real-time RT-PCR assay that has been described previously [15]. Results were expressed as cDNA equivalents per milliliter of plasma.

The Bio-Rad NS1 Ag Strip and SD Dengue Duo rapid tests were provided by Bio-Rad (Hercules, CA) and Standard Diagnostics (Kyonggi-do, Korea) respectively and were performed according to the manufacturer's instructions. Each plasma sample for assessment was tested on both rapid tests in parallel. Each assay strip was independently assessed after the incubation time suggested by the manufacturer by the technician conducting the test and by a 2^nd ^analyst who was blind to the first assessment. Discordant results were referred to a 3^rd ^analyst whose decision was final. The analysts performing and scoring the assays were blind to the reference assay results and to any clinical information on the patients.

### Analysis and Statistics

Concerning the SD Dengue Duo rapid test and for comparative purposes only, the IgM and IgG parameters were included in the interpretation of the test in some analyses. The statements "NS1 or IgM" and "NS1 or IgM or IgG" were then used and should be understood as if at least one of these parameters (i.e. NS1, IgM or IgG) is positive the sample is considered as positive. However, the detection of IgM and/or IgG in the rapid test is not sufficient for a definitive diagnosis of dengue.

All statistical analysis was performed using Intercooled STATA version 9.2 (StataCorp, TX). Significance was assigned at *P *< 0.05 for all parameters and were two-sided unless otherwise indicated. Uncertainty was expressed by 95% confidence intervals. Categorical variables between groups were compared by Fisher's exact test. The *t*-test was used for continuous variables.

## Results

### Characteristics of the study population

The characteristics of the study population (n = 292 cases) that contributed acute plasma to the test panel is shown in Table 1. The panel of dengue cases (n = 245) were consecutively enrolled, qRT-PCR positive patients in the DENCO study (see M&M). The median duration of illness prior to the test plasma sample being collected was 3 days (range: 1-6). There were 47 patients in whom there was no virological or serological evidence of acute or recent dengue in paired plasma specimens.

**Table 1:** Baseline table summarizing the characteristics of the patient population that contributed to the sample panel

Variable	Confirmed dengue (n = 245)	Other febrile illness (n = 47)
	N (%) or Median (range)	N (%) or Median (range)

Male sex	141 (57.8%)^a^	25 (55.6%)^b^

Age (years)	12 (1-49)^a^	9 (2-53)^b^

Day of illness	3 (1-6)	2 (1-6)^b^

**Infecting serotype and ** **serological status**		
**DENV-1**	**138 (56.3%)**	
Primary	49 (35.6%)	
Secondary	87 (63.0%)	
Indeterminate	2 (1.4%)	
**DENV-2**	**91 (37.61%)**	
Primary	9 (9.9%)	
Secondary	81 (89.0%)	
Indeterminate	1 (1.1%)	
**DENV-3**	**16 (6.6%)**	
Primary	8 (50.0%)	
Secondary	8 (50.0%)	

^a^sex and age was unknown in 1/245 acute dengue patients

^b^sex, age and day of illness was unknown in 2/47 patients with other febrile illness

### Sensitivity and specificity of NS1 tests versus reference algorithm and qRT-PCR

Bio-Rad and SD Duo NS1 rapid tests were equally sensitive for the diagnosis of acute dengue relative to the reference qRT-PCR test (Table 2; Bio-Rad 61.6% *vs *SD Duo NS1 62.4%, *P *= 0.93). The specificity of both NS1 tests was 100%, albeit the number of patients who had no evidence of acute or recent dengue was small (n = 47). Inclusion of the IgM parameter in the interpretation of the SD Duo test significantly increased its diagnostic sensitivity (Table 2; 75.5% *vs *62.4%, *P *= 0.0024). Inclusion of the IgM and the IgG parameters in the interpretation of the SD Duo test further increased the diagnostic sensitivity over NS1 alone (Table 2; 83.7% *vs *62.4%, *P *< 0.0001) but came at a cost of reduced specificity (100% for NS1 alone or NS1/IgM versus 98% for NS1/IgM/IgG).

**Table 2:** Sensitivity and specificity, positive and negative predictive values of each assay against the gold standard algorithm

Assay parameter	Patients (n =)	Acute Dengue cases (n =)	Number positive in rapid test (n =)	Sensitivity % (95% CI)	Specificity % (95% CI)	PPV % (95% CI)	NPV (%) (95% CI)
BR NS1	292	245	151	61.6 (55.2 - 67.8)	100 (93.8 - 100)^a^	100 (98.0 - 100)^a^	33.3 (25.6 - 41.8)

SD NS1 alone	292	245	153	62.4 (56.1 - 68.5)	100 (93.8 - 100)^a^	100 (98.1 - 100)^a^	33.8 (26.0 - 42.3)

SD NS1 or IgM^b^	292	245	185	75.5 (69.6 - 80.8)	100 (93.8 - 100)^a^	100 (98.4 - 100)^a^	43.9 (34.3 - 53.9)

SD NS1 or IgM or IgG^b^	292	245	206	83.7 (78.4 - 88.1)	97.9 (88.7 - 99.9)	99.5 (97.3 - 100.0)	53.5 (42.4 - 64.3)

^a^one-sided, 95% Confidence Interval

^b^the detection of IgM or IgG in the rapid test is not sufficient for a definitive diagnosis of dengue and results are reported here for comparative purposes only

### Sensitivity of NS1 tests by day of illness

The sensitivity of NS1 tests alone was not significantly different between test samples collected within 3 days of illness onset versus those collected at a later time (Table 3). However, if the result of SD IgM and IgG parameters were included in the interpretation of the SD test then sensitivity was significantly improved in test samples collected after 3 days of illness onset (Table 3; *P *= 0.05).

**Table 3:** Sensitivity of the dengue RDTs in samples collected within 3 days of illness onset versus those collected at a later time

		BR-NS1	SD-NS1	SD NS1 or IgM^b^	SD NS1 or IgM or IgG^b^
**Status**	**Total (n =)**	**% Sensitivity (95% CI)**			

Test sample ≤ 3 days of illness	156	60.9 (52.8 - 68.6)	62.2 (54.1 - 69.8)	73.1 (65.4 - 79.9)	80.1 (73.0 - 86.1)

Test sample > 3 days of illness	89	62.9 (52.0 - 72.9)	62.9 (52.0 - 72.9)	79.8 (69.9 - 87.6)	89.9 (81.7 - 95.3)

*P *value^a^		0.7860	1.0000	0.2809	0.0498

^a^Fisher's exact test

^b^the detection of IgM or IgG in the rapid test is not sufficient for a definitive diagnosis of dengue and results are reported here for comparative purposes only

### NS1 sensitivity in primary or secondary infection

**Figure 1:**
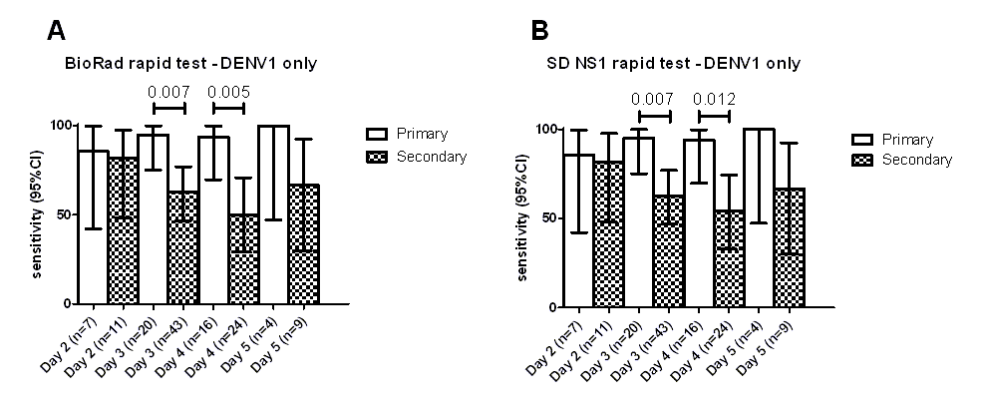
**NS1 sensitivity of Bio-Rad and SD rapid tests in relation to primary and secondary serological status and day of illness in DENV-1 infection**. Shown is the sensitivity of NS1 detection in the test sample according to primary and secondary serological status and day of illness for **A) **Bio-Rad NS1 test and **B) **SD NS1 test.

NS1 detection rates with both RDTs were significantly lower in patients with secondary dengue than primary dengue (Table 4). Interestingly, inclusion of the IgM and IgG parameters in the interpretation of the SD Duo test significantly increased the diagnostic sensitivity over NS1 alone in patients with secondary dengue (Table 4). The overall difference in sensitivity between primary and secondary dengue was not associated with the illness day at the time of testing (primary dengue, mean day of illness: 3.30 days versus secondary dengue: 3.26 days, *P *= 0.71). Reduced sensitivity was also not associated with viraemia levels between primary and secondary dengue cases (log10 mean viraemia for primary: 7.04 versus for secondary 6.78, *P *= 0.29). In a more stratified analysis, NS1 sensitivity was also higher in DENV-1 infected patients (where the sample size was highest) with primary dengue compared to secondary dengue at all time points of acute illness, (Figure 1A and 1B).

**Table 4:** Sensitivity of dengue RDTs in patients with primary and secondary serological profiles

		BR-NS1	SD-NS1	SD NS1 or IgM^b^	SD NS1 or IgM or IgG^b^
**Status**	**Total (n =)**	**% Sensitivity (95% CI)**			

Primary dengue	66	80.3 (68.7 - 89.1)	80.3 (68.7 - 89.1)	83.3 (72.1 - 91.4)	83.3 (72.1 - 91.4)

Secondary dengue	176	55.1 (47.4 - 62.6)	56.3 (48.6 - 63.7)	72.7 (65.5 - 79.2)	84.1 (77.8 - 89.2)

*P *value^a^		0.0003	0.0006	0.0951	0.8472

^a^Fisher's exact test

^b^the detection of IgM or IgG in the rapid test is not sufficient for a definitive diagnosis of dengue and results are reported here for comparative purposes only

A possible basis for reduced sensitivity in secondary dengue is that NS1, along with other viral antigens, is less likely to be available for detection when a substantial level of DENV-reactive IgG is present. To explore this further, we analyzed NS1 detection sensitivity in the context of DENV-reactive IgG and IgM status as defined by the reference ELISA in the test sample. The presence of measurable DENV-reactive IgG (in the GAC ELISA) in the test sample was associated with a significant reduction in NS1 sensitivity in the SD assay (Table 5). On the contrary, the presence of measurable DENV-reactive IgM (in the MAC ELISA) but not IgG (in the GAC ELISA) in the test sample was associated with a significant increase in NS1 sensitivity relative to seronegative test samples (Table 5).

**Table 5:** Sensitivity (95% CI) of SD NS1 RDT assay in the presence or absence of measurable DENV-reactive IgG or IgM detected by ELISA in the test sample

Status^a^	IgG+ & IgM-	IgG+ & IgM+	IgG- & IgM-	IgG- & IgM+
**% Sensitivity (95% CI)**	14.3 (4.0 - 32.7)	31.7 (18.1 - 48.1)	67.9 (58.2 - 76.7)	91.4 (82.3 - 96.8)

**Confirmed acute Dengue cases (n =)**	28	41	106	70

***P *value^b^**	0.1546		0.0001	0.0002

^a^according to the MAC and GAC reference ELISAs.

^b^Fisher's exact test

### NS1 sensitivity in relation to viraemia levels

**Figure 2:**
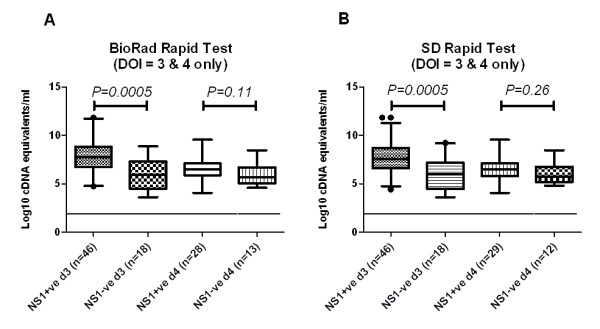
**Viraemia by NS1 status in DENV-1 infected patients samples at 3 and 4 days after illness onset**. Shown is the median (interquartile range, 95% range of data, and outliers) viraemia level in NS1 positive and NS1 negative patients with the same illness duration (3 and 4 days) tested by **A) **Bio-Rad NS1 test or **B) **SD Duo test. The limit of detection of the assay is shown with a dashed line. Viraemia levels were significantly higher in NS1 positive patients relative to NS1 negative patients (*t*-test) at day 3 of illness.

**Figure 3:**
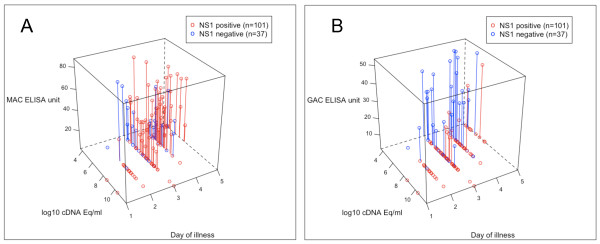
**NS1 status (with Bio-Rad rapid test) in relation to MAC and GAC ELISA results, viraemia and day of illness in DENV-1 infection**. Shown are **A) **MAC or **B) **GAC ELISA units, viraemia level and day of illness in NS1 positive and NS1 negative patients.

We hypothesized that viraemia levels would be associated with the detection of plasma NS1. Accordingly, in DENV-1 patients with equivalent illness durations (3 and 4 days of illness) viraemia levels were significantly higher (*P *= 0.0005) in patients who were NS1-positive versus those who NS1 negative in both tests at day 3 (with Bio-Rad and SD rapid test: log10 mean viraemia = 7.71 versus log10 mean viraemia = 6.11 respectively) (Figure 2A and 2B). To place NS1 detection in the context of both viraemia and serological response, individual test results for the Bio-Rad NS1 rapid test in DENV-1 infected patients were plotted against corresponding viraemia and serological responses in the same test sample (Figure 3A and 3B). These graphs illustrate the dynamic relationship between the detection of NS1, time since illness onset and in particular, the interfering effect of DENV-reactive IgG in the test sample.

## Discussion

In the present study we showed that two different commercially available lateral flow RDTs, the Bio-Rad NS1 Ag Strip and SD Dengue Duo, have similar sensitivities (61.6% *vs *62.4% respectively) for the detection of NS1 in plasma from RT-PCR positive patients. The factors negatively influencing the detection of NS1 included the presence of DENV-reactive IgG in the test sample and the presence of secondary infection. The inclusion of the IgM test in the SD Dengue Duo provides for greater sensitivity (75.5%) without compromising specificity. The reasonable sensitivity and specificity of the SD Dengue Duo suggests it warrants additional prospective evaluations.

There is, as yet, no dengue RDT that could be considered as providing a definitive diagnosis of dengue. At best, current tests provide a presumptive diagnosis for a patient with a dengue like illness. A recent, TDR/WHO sponsored multi-centre evaluation of anti-dengue virus IgM rapid tests highlighted the very modest specificity of several commercially available IgM rapid tests, particularly in samples from patients with other infections, with malaria being a major confounder [16]. As acknowledged by the authors of the evaluation study, specificity issues must be considered in the context of the setting in which a particular test is to be used [16]. For example, in SE Asia there is no Yellow Fever virus transmission and in many urban settings there is no malaria transmission that could serve to limit the accuracy of a dengue RDT. The challenge then is to identify the best test for a particular setting and this is best done by accumulating more systematic data across a range of patient populations in different health care settings. Decision-tree based algorithms, as suggested for selecting malaria rapid tests, could be helpful in this regard for dengue RDTs [17].

To the best of our knowledge this is the first side-by-side assessment of different NS1 RDTs. Our finding that they were equally sensitive for NS1 detection is encouraging and suggests other points of difference should be considered, e.g. ease of use, cost and stability at room temperature. The strength of the SD Dengue Duo is that the DENV IgM and IgG test windows provides an additional diagnostic investigation that complements NS1 detection. The inclusion of the DENV-IgM result in this current evaluation improved sensitivity without a reduction in the very high specificity associated with NS1 detection (100%). The inclusion of the IgG test result modestly further improved sensitivity. Caution is needed however as a positive IgM or IgG result alone could also represent infection anytime in the previous few months and should therefore be considered a presumptive diagnosis. The potential for reduced specificity was highlighted in that one patient with no laboratory acute evidence of dengue had a positive IgG test result alone. Nevertheless the use of IgM and IgG test parameters in a NS1 RDT is rational as it will likely provide improved presumptive diagnostic coverage towards the end of the acute illness when NS1 levels are declining but the DENV-specific IgM and IgG titres are climbing rapidly. The sensitivity and specificity of the IgM and IgG test components of the SD Dengue Duo have been described previously as part of the TDR/WHO assessment of dengue RDTs [16].

The presence of DENV-reactive IgG in the test sample, a relatively low viraemia and secondary dengue were the major factors associated with a negative NS1 finding on both tests. The bias of these RDTs towards patients with higher viraemia levels is probably a positive feature of these tests in that they are likely biased towards patients at risk for complications during their illness [18]. Somewhat surprisingly we did not replicate our previous observations in a different patient population that NS1 sensitivity by Bio-Rad rapid test was higher in the first 3 days of illness that at later times [11]. Differences exist between the two studies and these include- a) the first study included adults and b) the first study was done prospectively in real time with fresh plasma rather than frozen stored plasma. It is possible these factors might account for the differences between the study findings. It may also reflect chance differences associated with relatively small sample sizes. Similarly, we found greater NS1 sensitivity in samples where there was a measurable DENV-reactive IgM level, a finding inconsistent with our previous observations using ELISA based NS1 detection where the presence of IgM was not associated with higher or lower sensitivity [11]. The different findings might reflect chance differences in the patient population or differences between ELISA versus RDT.

A weakness of this study was that it was performed using a panel of stored plasma specimens and was heavily biased towards DENV-1, the most common serotype in circulation in Viet Nam since 2006. Similarly, assay performance and interpretation were performed by experienced lab analysts and not by clinicians "at the bedside". Different results may also have been obtained if an outpatient population, rather than a hospitalized set of patients, were used to generate the assessment panel used here. The evaluation panel is also biased in that all samples from dengue patients were RT-PCR positive. Despite these limitations, the current study provides a baseline in terms of sensitivity and specificity of these two RDTs for Vietnamese dengue patients and highlights virological and immunological factors associated with assay performance. Further prospective evaluations of both tests are warranted.

## Conclusions

Our findings suggest that the NS1 components of each test are specific tools for diagnosing acute dengue, though the sensitivity of both is influenced by the level of viraemia and host humoral immune response. The addition of an IgM/G component to the SD Dengue Duo significantly improved diagnostic sensitivity above NS1 testing alone.

## Competing interests

The authors declare that they have no competing interests.

## Authors' contributions

VT participated in the study design, the experimental work, the analysis and interpretation of the data and drafted the manuscript. HTTV participated in the experimental work and helped draft the manuscript. NVNQ participated in the study design, the experimental work and the analysis and interpretation of the data. CVVN, HTT, JF and BW participated in the study design, interpretation of data and revising of the manuscript. CPS conceived and designed the study and participated in the analysis and interpretation of the data and writing of the manuscript. All authors have read and approved the final manuscript.

## Pre-publication history

The pre-publication history for this paper can be accessed here:

http://www.biomedcentral.com/1471-2334/10/142/prepub
